# A Multi-scale Interfacial Investigation of Organic Friction Modifiers in Hydrocarbon

**DOI:** 10.1007/s11249-026-02137-w

**Published:** 2026-04-06

**Authors:** David Burgess, Peter J. Fryer, Ian McRobbie, Jacqueline Reid, Zhenyu Jason Zhang

**Affiliations:** 1 School of Chemical Engineering, University of Birmingham, Edgbaston, Birmingham, UK; 2 Innospec Ltd, Cheshire, Ellesmere Port, UK

**Keywords:** Nanotribology, Organic friction modifiers, Coefficient of friction (COF), Tribology

## Abstract

To link molecular characteristics and lubrication performance of Organic Friction Modifiers (OFMs), a multi-scale study was carried out to investigate the effects of functional groups and solvents on the tribological properties of two model OFMs, namely palmitic acid and pentadecylamine. It was observed that the solvent composition has a profound effect on the surface adsorption and the interfacial adhesion force of the OFMs, showing vastly different values of adsorption kinetics and surface coverage. It must be highlighted that the presence of water within the hydrocarbon has a considerable impact on the lubrication performance of OFMs. We were able to quantitatively correlate the molecular properties such as binding force and adsorption kinetics of the OFM with their macroscopic testing results satisfactorily, suggesting that the static Coefficient of Friction (COF) is governed by the properties of the film formed in a thermodynamic equilibrium, whilst the dynamic COF is related to the interfacial forces at the surface during sliding, and finally the wear on the solid surface is related to ability of the OFMs to rapidly re-adsorb on the solid substrate once removed.

## Introduction

Modulating surface characteristics to reduce boundary friction in hydrocarbon has been a highly researched topic since 1920’s when Langmuir [1] reported that natural plant and animal fats can be used to reduce the surface forces in the boundary lubrication regime. Bowden and Tabor [2] subsequently developed a model to explain how these molecules, commonly known as Organic Friction Modifiers (OFMs) can alter the surface properties, reducing friction and wear. OFM molecules often contain a polar headgroup that interacts with the steel, likely via hydrogen bonding [2], and a hydrophobic tail group that solvates in the non-polar solvent, which separates the surface asperities from contact.

Much of the research concerning OFM development or understanding involves a variety of friction testing rigs to evaluate the friction reducing ability [3, 4], of which the most prevalent ones for evaluating boundary friction are techniques such as Scuffing Load Ball-on-Cylinder Lubricity Evaluator (SL-BOCLE) and High-Frequency Reciprocating Rig (HFRR). A notable example of macroscale testing for friction and wear is in the fuel industry where HFRR is used as the ISO standard method for evaluating the lubricity of a fuel [5, 6]. Some studies concerning the effectiveness of HFRR suggested that it could be an aggressive method because the initial contact pressures are very high, which deforms the solid substrate before the measurement is commissioned [5, 7]. However, studies demonstrated that HFRR is superior to other benchtop macroscale testing methods in distinguishing the lubrication performance of OFMs [6, 8, 9].

Whilst macroscopic testing rigs can provide a useful lubricity assessment of OFMs, they do not necessarily offer the knowledge concerning the detailed molecular characteristics that govern the mechanisms responsible for reducing friction and wear. Previous studies confirmed that the overall friction performance measured on the macroscale is a function of several factors, including OFMs chemistry, the condition and material of the substrate, the solvent, and interfacial phenomena along with many more factors [10].

More recently, OFMs were investigated using nanotribological techniques to better understand the molecular characteristics and interfacial behaviour of OFMs that influence oil lubricity [11–19]. Here, the synergistic or antagonistic effects of OFMs on interfacial lubrication, demonstrated with amines of different degree of saturation mixed with either glycerol monooleate (GMO) or oleic acid, were reported by Fry and colleagues [20]. To investigate the effect of surface adsorption [21], the same group reported that the thicker OFM layers result in narrower, rougher wear tracks than the wider, smoother tracks observed with thinner OFM layers, highlighting the apparent effect of OFM is dependent on the stage of the rubbing test: initially on friction, and subsequently on surface damage. When it comes to the effect of molecular characteristics of OFMs, Nalam and colleagues examined fatty amines with varied degree of branching using QCM and atomic force microscopy (AFM) [22], reporting that the OFMs could re-adsorb onto the solid substrate after being removed during a tribological process. A counterintuitive result was reported that the more mass adsorbed onto the surface, the greater the Coefficient of Friction (COF), despite the follow-up simulation work shows that this is due to the very sharp AFM tip penetrating and being confined within the adsorbed OFMs layer [22, 23].

Functional groups are critical for the molecular design of OFMs, which was demonstrated in a recent work that combines both experimental and computational investigation [24], in which nine organic compounds were studied. It was found that OFM molecules with high adsorption energy are more likely to adsorb on substrates and form a vertical monolayer, which can maintain a regular molecular brush structure during friction and ensure a low friction coefficient. Molecular simulation studies [19, 25] by Bonnaud and colleagues reinforce the same message using amine-based organic additives in base oil at a model of steel surface.

Quality and characteristics of the hydrocarbon, in which OFMs are present, are equally important. Hexadecane is commonly used in laboratory studies concerning OFMs [18, 22, 26, 27], although there are different views regarding the influence of solvent on OFM performance. Jaishankar and co-workers reported that there is a significant decrease in the adsorbed mass of stearic acid in heptane compared to hexadecane [28]. However, it was found, by a different group, that there is little change in adsorption comparing hexadecane to a heavily branched isomer [13]. Solubility of OFMs in the base oil, as demonstrated in a recent work by Gu and colleagues using cetyl alcohol as a model additive [29], reflects the importance of solvation free energy on friction-reduction ability. It must be highlighted that the hydrocarbon deployed in real-world applications are much more complex than pure saturated alkanes, consisting of hundreds of compounds as well as water. Therefore, it is of great importance to understand the impact of solvent quality on the OFM performance and how the complex real-world hydrocarbon mixtures interact with the OFMs [30, 31].

A comprehensive suite of experimental, theoretical, and computational approaches have been implemented to investigate the interfacial characteristics of OFMs, demonstrating the possibilities to decouple the factors reviewed above in establishing insights at meso- and nanoscale. However, relating these phenomena to the macroscale friction performance remains a challenging task, particularly relating molecular characteristics of the OFM and the wear results in the context of the interfacial dynamics [22, 32]. The aim of the present study is to establish a multi-scale understanding of the tribological characteristics of OFMs in model fuels, which integrates molecular understanding on factors such as molecular architecture, interfacial kinetics, and solvent quality, with the overall macroscopic frictional behaviour. This was accomplished using nanotribological techniques to investigate the molecular properties of palmitic acid and pentadecylamine in hydrocarbon, including hexadecane and two complex hydrocarbon, which allows us to establish the effect of hydrocarbon composition on the tribological performance of the additives. Correlation of macroscopic tribological behaviour with molecular architecture was subsequently established with the results generated by macroscale friction techniques, which offers insights on potential strategies in optimising the design of OFMs.

## Materials and Methods

### Materials

Two model hydrocarbon samples were kindly provided by Innospec Ltd, of which the ratios of saturates, olefins, and aromatics are summarised in Table 1. A benchmark, *n*-hexadecane anhydrous (99%, Sigma-Aldrich, UK) was included. Palmitic acid (> 99%), pentadecylamine (> 96%), 12-mercaptododecanoic acid (96%), 11-amino-1-undecanethiol hydrochloride (99%), trifluoroacetic acid (99%), and triethylamine (99%) were purchased from Sigma-Aldrich (Dorset, UK) and used as received. Chloroform (99%), toluene (anhydrous, 99.8%), acetone (HPLC grade, 99.9%), ethanol (absolute, 99%), sulphuric acid (Puriss p.a. 97%), and hydrogen peroxide were purchased from Fisher Scientific (Loughborough, UK) and used as received. Concentration of the model OFMs was kept at 150 mg L^−1^.

**Table 1:** Table showing the relative compositions of the hydrocarbon samples being investigated

Solvent	Saturate content/%	Olefin content/%	Aromatic content/%	Kinematic Viscosity/mm^2^ s^−1^	Density/g cm^−2^
*n* – Hexadecane	100	0	0	4.0	0.773
Saturated oil	100	0	0	3.9	0.82
Aromatic oil	82.6	1.5	15.9	4.2	0.84

The steel substrate used for testing was grade AISI 52100 (BSI 100Cr6), purchased from PCS Instruments (UK). The steel used in the atomic force microscopy testing is a disc of 10 mm in diameter and a surface roughness of 0.02 µm (*R*_a_). The steel balls used for wear testing are 6 mm in diameter and have a surface roughness of 0.06 µm (*R*_a_). Stainless steel-coated QCM sensors (QSX203) were purchased from Biolin Scientific (Sweden) to provide a consistency with the other measurements.

Interfacial adhesion and lateral force measurements by the atomic force microscope were carried out using a diamond-like carbon coated cantilever (Tap150DLC, Budgetsensors, Bulgaria) with a nominal spring constant of 5 N m^−1^. For the binding force measurements, chemically functionalised gold-coated AFM cantilevers (PNP-TR-Au, Nanoworld, Switzerland) with a nominal spring constant of 0.32 N m^−1^ were prepared and used.

### Surface Cleaning

A rigorous cleaning procedure is required to ensure a consistently clean surface was used in all measurements [18]. All steel specimens were cleaned by following the ISO standard procedure for the preparation of steel for HFRR testing (ISO 12156–1). The steel specimen was sonicated in toluene for 15 min initially, followed by sonication in acetone for 15 min, dried under a stream of nitrogen gas, and finally placed under UV-Ozone (Ossila, UK) for 15 min to ensure the surface is free from organic contaminant. The QCM sensors were cleaned following the same process without sonication to avoid potential physical disruption to the sensor surface.

### Preparation of Self-Assembled Monolayers (SAMs)

SAMs were prepared on gold-coated cantilevers using 11-mercaptoundecanethiol hydrochloride to form an amine-terminated SAM and 12-mercaptododecanoic acid to prepare a carboxylic acid terminated SAM. The SAMs were prepared following the protocol reported previously [33].

The glassware was all cleaned using a piranha solution consisting of 70% sulphuric acid and 30% hydrogen peroxide before being rinsed with copious amount of water and subsequently placed in a clean oven to dry overnight. The ethanolic solution of thiol was prepared at a concentration of 1 mmol dm^−3^ and the gold-coated cantilevers were submerged in the thiol solution for 20 h and wrapped in foil to avoid sunlight. The cantilevers were then rinsed with ammonium solution and ethanol to remove any of the unbound thiols from the surface and dried under a stream of N_2_ gas. The cantilevers were used on same day to measure the surface binding, ensuring quality with minimum degradation of the formed SAMs.

### Atomic Force Microscopy

A dimension 3100 (Veeco, UK) was used to quantify the interfacial adhesion force and binding force in the solvent of interest by keeping an adequate amount of the solvent between the cleaned steel substrate and the AFM cantilever for 40 min, based on the adsorption kinetics measured by QCM, which allows sufficient time for the additives to adsorb on the surface and the AFM tip. A total 100 force curves were acquired with a maximum applied force of 25 nN in a 10 × 10 grid over a 1 µm^2^ area, which was repeated five times at different locations of the surface to obtain the mean interfacial adhesion and the mean binding forces.

When measuring friction with the AFM, a diamond-like carbon (DLC) AFM tip was used. This is due to the enhanced durability of the AFM tip when applying pressure into the surface and sliding along the substrate to ensure the friction experienced is from the surface interactions and not the degradation of the AFM tip. To measure the friction with AFM, the lateral deflection sensitivity of the cantilever was calibrated using a TGF-11 calibration grid containing a set of inclining and declining slopes at different gradients, following the method reported by Ogletree and colleagues*.*[34] Once the lateral deflection was calibrated, the cantilever slides across the surface at a set normal load over 1 µm at a speed of 1 µm s^−1^, during which the friction signal was acquired by taking the Trace-Mean-Retrace (TMR) value. Ten values of the TMR were taken for each normal load and the average was taken. The normal load was then increased and the new TMR was taken, from which the lateral force was calculated. This was repeated for a variety of normal loads. The measurement was carried out in triplicates to ensure repeatability [35, 36].

### Quartz Crystal Microbalance

An OpenQCM Q^−1^ (Italy) with a PTFE flow cover and a chemically resistant Viton tubing was used to measure adsorption and desorption, for which the flow was controlled using a high accuracy microflow peristaltic pump (Ismatec, UK). The blank solvent being investigated was flowed over the cleaned stainless steel sensor at a flow rate of 55 µL min^−1^ for 6 h until a stable baseline was achieved, i.e. no change in frequency greater than 5 Hz over the course of 5 min. Once the frequency was stable, the tubing was switched to the solvent containing the additives being investigated and was run until a plateau was observed in the frequency to ensure a maximum of adsorption had been observed i.e. no change in frequency greater than 5 Hz over the course of 5 min.

### Langmuir Trough

A Langmuir trough was used to investigate the surface packing and compression behaviour of the model additives at water/air interface. The Langmuir trough (G2 microtrough, Kibron, Finland) was cleaned using chloroform and filled with ultrapure water. A Wilhelmy plate was cleaned by passing through a flame several times and rinsed with ultrapure water before being placed, with a hanging meniscus, on the water in the trough to measure the surface pressure that represents the difference between the surface tension of the pure subphase (*γ*_0_) and the surface tension of the monolayer-covered surface (*γ*). The barriers were initially reduced to full compression to check the cleanliness of the water sub-phase. If a change in the surface pressure was observed during the compression of a pure water sub-phase, the surface is aspirated to remove any surface contaminants and the barriers compress again—this was repeated until a maximum change in surface pressure of 0.5 mN m^−1^ was achieved.

A total 30 µl chloroform containing OFMs at 1 mg ml^−1^ was pipetted onto the water sub-phase. After waiting 15 min for all of chloroform to evaporate, the measurement was ready to proceed. The barriers compress at a speed of 5 mm min^−1^ and run a full compression and the change in surface pressure was measured with the changing area of the 2D sub-phase.

### Surface Tension

To measure the surface tension of the liquid, a custom-build set up was used, whereby a fibre optic light source (Dual Fiber unit LED, Germany) was shone through a light diffuser (DG20-600-MD, Thor labs, UK) on the measurement stage, of which the image was captured by a camera provided by First Ten Angstroms (UK).

For the actual measurement, a syringe filled with the organic solvent being investigated was place between the light and the camera, and a 21 GA blunt tipped needle was placed on the syringe. A known volume of liquid was syringed out and a picture of the droplet formed was taken, of which the droplet shape was analysed using the First Ten Angstroms software.

### Water Content Determination

The water content in the hydrocarbon samples was measured using a KF Ti-touch Karl Fischer titrator (Metrohm, UK). A small quantity of known mass (between 0.5 and 1 g) was pipetted into the titration equipment and then the water content was measured.

### Rotating Wear Tester

A rotating wear tester (Forceboard, Sweden) was used for the macroscale friction measurements, during which a steel ball of radius 3 mm was used as the top contact onto a steel disc of radius 250 mm. A weight of 1 kg was applied to the beam, generating a vertical load of 6.2 N, which results in a mean Hertzian contact pressure of 0.8 GPa. Due to the high pressures and low viscosities the testing is evaluated in the boundary lubrication regime where OFMs are deemed to be most effective. Testing liquid of 100 ml was subsequently added to the reservoir so that the bottom of the rotating steel disc was submerged in the hydrocarbon samples. The disc was rotated at a speed of 240 rpm for 15 min, corresponding to a sliding speed of 1 m s^−1^. The lateral friction and COF were measured over the course of the measurement, from which both static COF (when the rotating disc is at a stop after rotating) and dynamic COF (during the sliding) were acquired.

The steel ball was then put under an AxioScope A1 microscope (Zeiss, Germany) and the corresponding wear width was measured using the Zeiss software. The averaged COF and wear scar diameter were taken over five samples.

## Results and Discussion

### Physical Properties of the Hydrocarbon Specimen

**Fig. 1:**
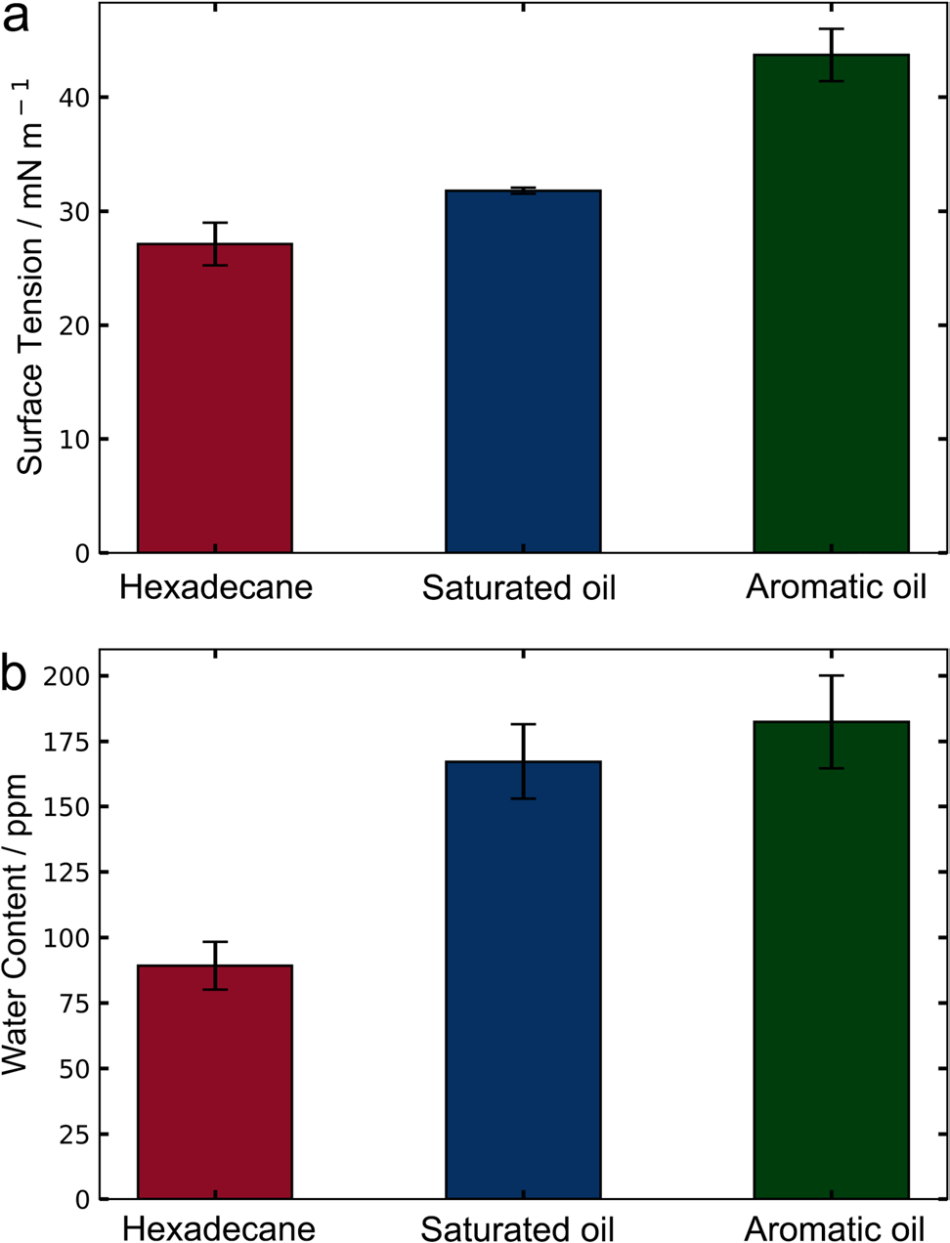
**a** Surface tensions and **b** water contents of hexadecane and two commercial fuels, saturated oil, and aromatic oil

Pendant droplet measurements were carried out to evaluate the surface tension of the hydrocarbon samples used in the present work, of which the values are presented in Fig. 1a. The surface tension of hexadecane, ~ 27.5 mN m^−1^, agrees with the values (27 – 28 mN m^−1^ at 293 K) reported in the literature [37, 38], which validates the method used, too. The differences observed in surface tension values are probably because the saturated oil is a mixture of compounds ranging in chain lengths as opposed to a pure hexadecane. It is worth noting that the differences in hydrocarbon compositions likely plays a significant impact on how OFMs behave in the bulk and at the interfaces. This is because the driving force for adsorption to the surface in such a non-polar environment is most likely due to the polar interactions such as hydrogen bonding between the functional headgroup of OFMs and the steel surface. The introduction of polarity to the organic solvent will very likely compete for surface binding sites available, and therefore compromise the interfacial lubrication performance of the OFMs [39, 40].

One of the most abundant but difficult to avoid impurities in a commercial hydrocarbon is water. Depending on the chemical nature, commercial products would absorb moisture from the surrounding ambient at different rates. The potential influence of water molecules in the hydrocarbon at concentrations similar to that of the additives could be significant in competing for the binding sites available on the steel surface. The water contents in the three hydrocarbon specimens tested in the present work, measured by Karl Fischer titrations, are shown in Fig. 1b. Hexadecane has a significantly lower water content of 90 ppm, in comparison with the saturated oil that has a similar composition but a water content of 170 ppm. The aromatic oil has a slightly increased water content of around 180 ppm, which is consistent with literature [41, 42]. The variation in water content could be the cause of the observed differences in the surface tension, as the increased presence of water will increase the polar component of the oils surface tension, thus increasing the overall surface tension. Whilst the saturated oil and the hexadecane have a similar composition, the behaviour of the OFMs in these oils could be profoundly different due to the presence of water. Nanotribology was chosen as the method to investigate how the molecular architecture and solvent impact the adsorption processes, and the forces at the interface of the OFMs film formed in thermodynamic equilibrium.

### Evaluating Interfacial Adhesion and Surface Binding

**Fig. 2:**
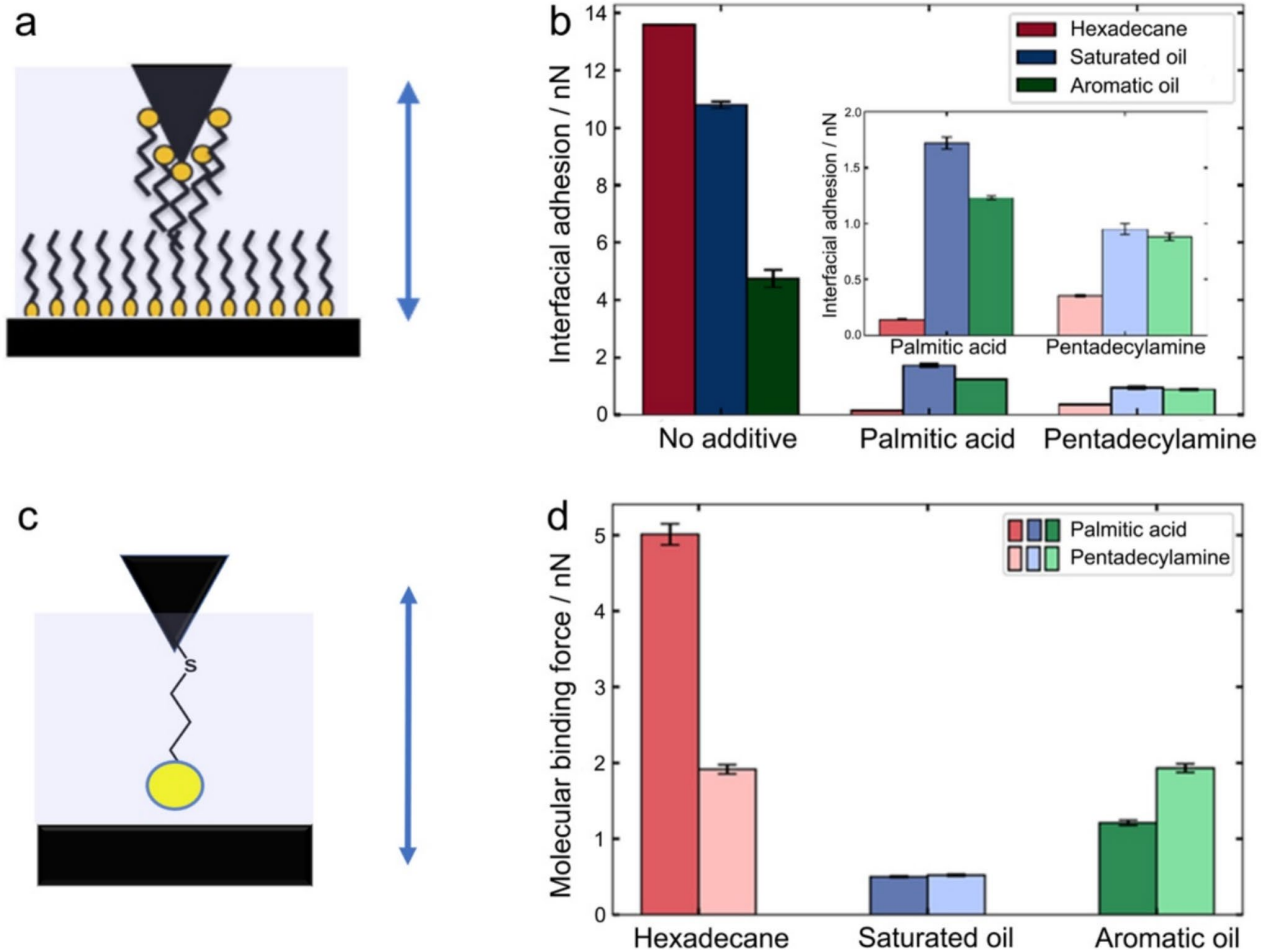
**a** Schematic diagram showing the interfacial adhesion measurements. **b** Interfacial adhesion of the different additives in different hydrocarbon samples. **c** Schematic showing the molecular binding measurement configuration. **d** Molecular binding data of the different headgroups in three different hydrocarbon samples. Note that the shaded regions in 2a and 2c indicate the presence of hydrocarbon

Interfacial adhesion between a DLC-coated AFM tip and a steel surface, with or without the presence of the two representative OFMs, was quantified using AFM-based force spectroscopy. Schematic diagram demonstrating the interfacial adhesion measurement is shown in Fig. 2a, with the corresponding values presented in Fig. 2b. It was observed that the interfacial adhesion on steel with no additives present is significantly greater than those with the presence of the OFMs, which reinforces the well accepted mechanism that the surface adsorbed OFM molecules could greatly reduce the adhesive interactions at the interface [22]. This would in turn decrease the interfacial friction, improving the lubricity of the hydrocarbon. Without the adsorbed additives, surface adhesion was found to be 14, 11, and 5 nN in hexadecane, saturated oil, and aromatic oil, respectively, as shown in Fig. 2b. The changes in the surface tension of these hydrocarbon samples, shown in Fig. 1a, are likely to be the cause for the differences in interfacial adhesion measured. Surface tension of a liquid reflect the magnitude of the interaction between a liquid and a solid—the large surface tension of the aromatic oil could be attributed to its increased content of water molecules that likely interact favourably with the steel, which results in the lowest interfacial adhesion force between all three solvents (without the addition of OFMs) tested. The hexadecane has the smallest surface tension and exhibits the largest interfacial adhesion force. The observation highlights that the interfacial energy, reflecting the affinity of the solvent with the solid surface, could considerably influence the lubricity of the oils without additives present. When there is no OFM present in the hydrocarbon, the one with the highest surface tension will have the lower interfacial adhesion with the steel substrate.

Once the OFMs were introduced in the hydrocarbon, a different trend was observed. The two model OFMs, palmitic acid and pentadecylamine, were chosen due to their same hydrocarbon chain but contrasting functional groups (carboxylic acid *versus* amine). It therefore can be assumed that any differences observed in the interfacial adhesion force are either due to the packing and configuration of the adsorbed OFM film (same OFM but varied solvent) or the nature and magnitude of the binding (same solvent but different OFMs). The inset of Fig. 2b shows an amplified view of the interfacial adhesion with the presence of OFMs. Both palmitic acid and pentadecylamine result in the lowest interfacial adhesion on the steel substrate in hexadecane, implying that a densely packed OFM film was formed at the interface. Both additives in aromatic oil show the second highest interfacial adhesion force, which could be due to the presence of aromatics in the fuel being less likely to sufficiently solvate the alkane tail groups at the interface, hence increasing the interfacial adhesion.

For palmitic acid, the greatest interfacial adhesion was observed in the saturated oil, as shown in the inset of Fig. 2b, suggesting that the least packed film was formed. This is unexpected given that the saturated oil and the hexadecane are similar in terms of composition. It is probable that the water molecules in the saturated oil negatively impact the adsorption and packing of the palmitic acid molecules, resulting in a less well packed film. Such effect was also noted in the aromatic oil, to a less extent, though. Pentadecylamine shows a similar trend but on a reduced magnitude, implying that the amine functional group is less influenced by the presence of water when adsorbing on the steel surface, suggesting that the amine headgroup outcompetes the water to the surface better.

To investigate the effect of the headgroups and solvents on the binding of OFMs to the steel surface, chemical force microscopy was carried out. AFM cantilevers were functionalised with either acid- or amine-terminated SAMs, and force curves were acquired against a steel surface whilst submerged in the hydrocarbon. The nature of such measurement is presented as a schematic diagram in Fig. 2c. It quantifies the magnitude of the binding force of the functional groups to the steel surface in the hydrocarbon samples of interest, of which the averaged values are shown in Fig. 2d. The strongest binding force (~ 5 nN) was observed with the −COOH SAM in hexadecane, which is several times greater than the values acquired in the other two specimen for the same functional group. The result suggests that palmitic acid molecules could bind to the steel surface most strongly in hexadecane, leading to a well packed surface adsorbed layer, which is evidenced by the minimal interfacial adhesion force (Fig. 2b). The binding force (~ 2 nN) of the -NH_2_ SAM in hexadecane is less than half of the values for the -COOH SAM, suggesting that the acid groups bind strongest to the steel in the neat hexadecane, with no other compounds other than ca. 75 ppm water.

The binding forces onto the steel substrate differ significantly for the two complex hydrocarbon samples: saturated oil and aromatic oil. The forces are weak, especially in the saturated oil where both functional groups exhibit low values of ca. 0.8 nN, although the amine-terminated SAM appears to bind slightly stronger to the solid substrate than the acid-terminated SAM in the aromatic oil. By changing from a neat compound (hexadecane) to complex oils, the surface binding force has changed significantly, demonstrating that the composition of the solvent has a profound effect on the surface binding. The quantitative data demonstrates that the compounds present in the complex hydrocarbon but not in hexadecane could inhibit the ability of the functional SAMs to interact with steel surface effectively, which is applicable for the surface adsorption process of OFMs. It is worth noting that the water present in the hydrocarbon could decrease the surface binding of the OFMs. Competition between water and headgroups of OFMs could create an antagonistic effect on the surface binding affinity, whereby additives might (i) bind to a thin water layer at the surface or (ii) preferentially binding with the water in the hydrocarbon, leaving less OFM molecules to interact with the surface.

### Single Asperity Friction Measurements

**Fig. 3:**
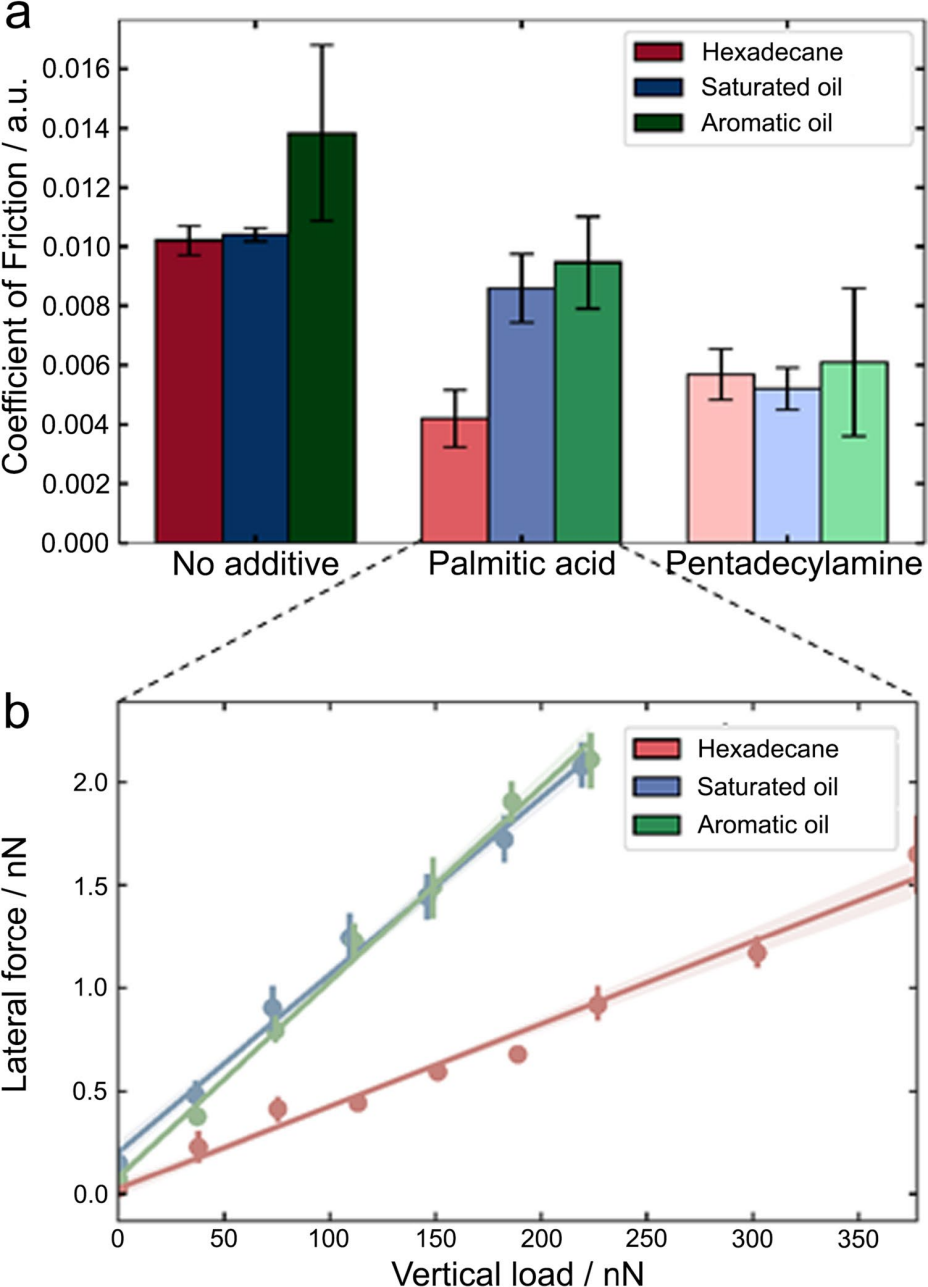
**a** COF values of the OFMs in different hydrocarbon samples, measured by nanotribology. **b** Representative friction-load plots acquired when palmitic acid was used in three samples, and the line of best fit used to calculate the COF

To further evaluate the effect of OFMs on the interfacial friction, specifically at single asperity, nanotribology measurements based on AFM were carried out, of which the results are shown in Fig. 3. It was observed that the friction-load plots are linear, suggesting that the contact follows Amonton’s law, which allows us to extract the COF quantitatively [43–45]. The averaged values of COF, acquired in three hydrocarbon specimen, with or without the presence of the two model OFMs, are shown in Fig. 3a. It was observed that all three hydrocarbon samples without OFMs exhibit the highest COF: both hexadecane and saturated oil showing a COF of ~ 0.010, whilst the aromatic oil results in the highest COF of ~ 0.014, with the largest variability. It is likely that some of the olefins and aromatics in the aromatic oil adsorb to the steel surface non-uniformly, causing a broad variation in the friction on the surface at the nanoscale. It is worth highlighting that the hydrocarbon samples with low surface tension produced low COF values, likely due to their ability to wet the solid surface [46, 47].

Once the model OFMs were introduced in the hydrocarbon, the COF values were reduced by 20–60%, which evidences the effectiveness of the surface adsorbed OFM molecules in decreasing interfacial adhesion and friction. For palmitic acid, the lowest COF was observed in hexadecane, whilst the values are similar in saturated oil and aromatic oil. The limiting effect of hydrocarbon on palmitic acid to lubricate the interface is likely due to the presence of water that could compete for binding groups available on the steel surface and formation of well packed palmitic acid film. In the aromatic oil, the more polar olefins and aromatics could be causing this interference. There is little variation with COF between different hydrocarbon with pentadecylamine additive, though, implying that the presence of water does not necessarily affect the friction performance of the pentadecylamine film. This is inconsistent with the interfacial adhesion data where the water content appears to increase the interfacial adhesion of the pentadecylamine film, albeit less so than the palmitic acid (Fig. 2a). We speculate that the trace amount of impurities in the solvent could have a more noticeable effect on the adhesion at the interface of the OFM layer than the nanoscale friction.

### Surface Adsorption of the Model OFMs

**Fig. 4:**
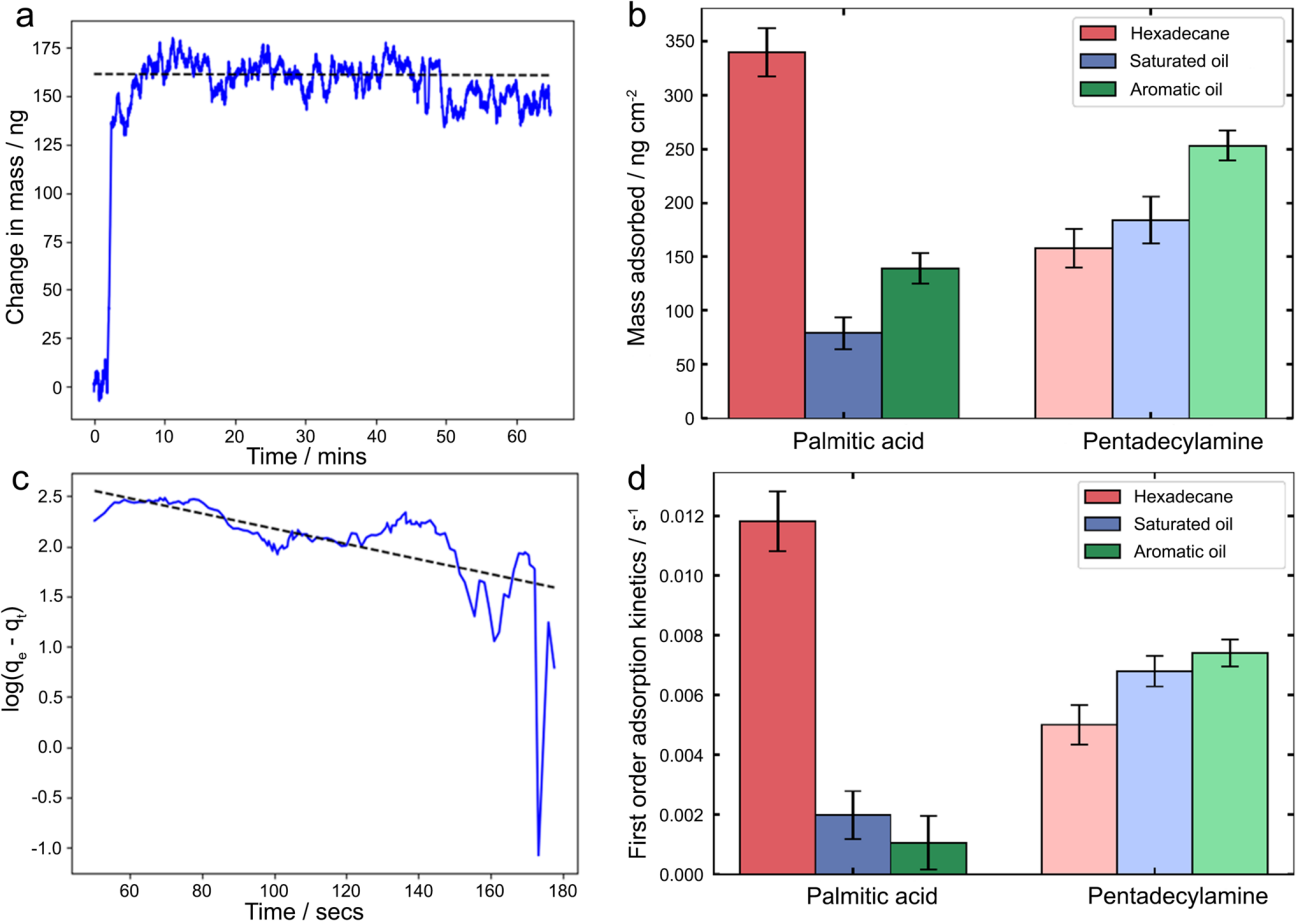
**a** A representative QCM data showing mass calculated as a function of time, acquired when pentadecylamine was introduced in hexadecane. **b** Maximum adsorbed mass onto the stainless steel surface for the OFMs in different hydrocarbons. **c** A representative plot demonstrating the calculation of the first-order adsorption kinetics. **d** First-order adsorption kinetics calculated for both OFMs in different hydrocarbon

Results from AFM-based measurements reveal both surface binding and adsorption of OFMs, and subsequent formation of the adsorbed film, are critical to their ability to lubricate a solid substrate, of which the kinetics need to be established [16, 22, 48]. This is particularly critical in real-world applications where oil additives operate in a dynamic process that involves transport of the additives from the bulk oil to the oil–surface interface, followed by surface adsorption of the additives onto the stainless steel surface. QCM was deployed to measure the adsorption processes of the model OFMs to the surface by monitoring the change in frequency of a stainless steel-coated QCM sensor over time. The adsorbed mass can be calculated from the change in resonate frequency of the sensor, using Sauerbrey equation (Eq. 1) [49].

1 $$\Delta f_{n} = - n\frac{{2f_{0,n}^{2} }}{{\sqrt {\mu_{q} \rho_{q} } }}\Delta m,$$

where $\Delta {f}_{n}$is the change in the resonant frequency at the $n$^th^ harmonic of the oscillating quartz sensor in Hz, ${f}_{0,n}$is the resonant frequency at at the $n$^th^ harmonic in Hz, ${\mu }_{q}$ is the shear elastic modulus of the AT-cut quartz in kg m ^−1^ s^−1^, ${\rho }_{q}$ is the density of the quartz in kg m^−3^ and $\Delta m$ is the areal mass of the film in kg m^−2^. Equation 1 can be simplified to Eq. 2:

2 $$\Delta m = - C\frac{\Delta f}{n},$$

where $C$ contains all the constants defined above and is a term known as the mass sensitivity constant that is 17.7 ng cm^−2^ Hz^−1^ for a 5-MHz QCM sensor. The measurements were carried out taking the 0th and 3rd overtones. A representative QCM data is shown in Fig. 4a, demonstrating the surface adsorption process as a function of time, followed by reaching equilibrium [50], from which the maximum mass adsorbed was extracted and shown in Fig. 4b. For palmitic acid, the maximum mass adsorbed (~ 330 ng cm^−2^) was observed in hexadecane, which is consistent with the interfacial adhesion data (Fig. 2b), confirming that more palmitic acid molecules adsorbed on the stainless steel surface and form a packed layer, in comparison to the other two complex hydrocarbon samples. QCM data suggest that there are considerably less palmitic acid molecules (less than 150 ng cm^−2^) adsorbed in the saturated and aromatic oil, which explains the increased COF values measured (Fig. 3a). The surface adsorption amount of pentadecylamine in hexadecane is approximately 150 ng cm^−2^, merely half of that for palmitic acid, which is in line with the interfacial adhesion, surface binding, and nanofriction results (Fig. 2, 3). There is a slight increase of the adsorbed mass in saturated oil and aromatic oil, though.

By fitting the mass change over time to a derivative of the Langmuir adsorption isotherm, the first-order adsorption kinetics could be obtained by fitting the initial portion, the first 200–300 s of the adsorption, to Eq. 3.[51]

3 $$Log_{10} \left( {Q_{e} - Q_{t} } \right) = Log_{10} \left( {Q_{e} } \right) - \frac{{k_{ads} }}{2.303}t,$$

where ${Q}_{\mathrm{e}}$ is the maximum mass adsorbed in ng, ${Q}_{\mathrm{t}}$ is the mass adsorbed at time $t$ in ng, ${k}_{\mathrm{ads}}$ is the first-order adsorption kinetics in s^−1^, and $t$ is the time in s.

A representative plot, presenting ${Log}_{10}\left({Q}_{\mathrm{e}}-{Q}_{\mathrm{t}}\right)$ as a function of $t$, is shown in Fig. 4c, from which the first-order adsorption kinetics was obtained from the gradient.^16, 22^ The calculated first-order adsorption kinetic values for both model OFMs in all three solvents are included in Fig. 4d. It was observed that palmitic acid shows the fastest adsorption kinetics in hexadecane (0.012 s^−1^) and the slowest adsorption kinetics in aromatic oil (0.001 s^−1^), which strongly suggests that water molecules interact with the stainless steel surface, inhibiting the adsorption of palmitic acid molecules to the surface, and potentially interact with palmitic acid in the bulk hydrocarbon too, which reduces the diffusion of the additives to the liquid/solid interface. Interestingly, pentadecylamine showed slow adsorption kinetics in hexadecane (0.005 s^−1^) but a slightly increased adsorption kinetics in the saturated oil and aromatic oil (both around 0.007 s^−1^), suggesting that the complexity of the hydrocarbon does not affect the adsorption of pentadecylamine in the same way as palmitic acid. The varying magnitude of the solvent effect on OFMs suggests the presence of polar molecules contained in the solvent could be more significant on the palmitic acid. However, this could create antagonistic competitive effects that are minimised or even synergistically assisting the surface adsorption of pentadecylamine.

### Molecular Packing of OFMs at Interface

**Fig. 5:**
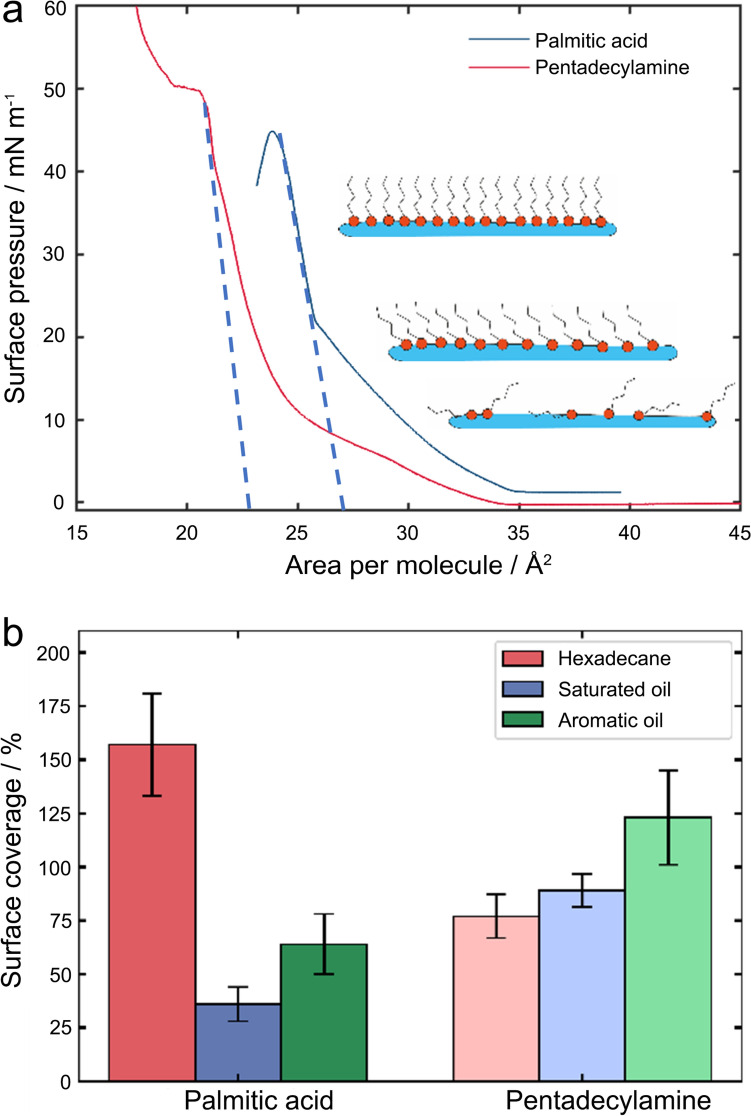
**a** Pressure–area isotherms of palmitic acid and pentadecylamine on a water sub-phase. **b** Calculated surface coverage of the OFMs on the stainless steel surface in different hydrocarbons

To better understand the interfacial configuration of pentadecylamine and palmitic acid in the surface adsorbed layer, a Langmuir trough was used to measure surface pressure as a function of surface area, which generates compression isotherms, of which a representative one is shown in Fig. 5a. It was found that both model OFMs remain in the 2D gaseous phase at around 35 Å^2^ per molecule, suggesting that the molecules were relatively spread out, and there was no change in surface pressure of the air–water interface.

As the surface area continues to decrease upon compression, the amphiphilic molecules scattered at the air–water interface start to come closer and enter the liquid condensed phase where the molecules begin to compress together, and the film slowly becomes more ordered with the decreasing surface area. This is shown by the slow increase in surface pressure from 0 mN m^−1^ to ca. 20 mN m^−1^.

With a further decreasing surface area, the surface pressure rises abruptly until it reaches the maximum (approximately 40–50 mN m^−1^), at which point the OFM molecules are in the 2D solid phase where they reach the maximum monolayer packing. The monolayer would collapse once the area is reduced beyond the 2D solid phase, with molecules forming micelles, bi- or tri-layers at the air–water interface, as evidenced by the dropped surface pressure. By extrapolating the linear portion of the solid phase to the X-axis (dotted lines in Fig. 5a), the limiting area per molecule for both palmitic acid and pentadecylamine were obtained. This is the area one molecule takes up under the maximal compression in the monolayer, commonly referred as the cross-sectional surface area [52, 53]. The cross-sectional surface area is 23 Å^2^ and 27 Å^2^ for pentadecylamine and palmitic acid, respectively, suggesting that a pentadecylamine molecule occupies less space when tightly packed together. With this information and knowledge of the total amount of mass adsorbed measured by the QCM, the surface coverage of the model OFMs on the stainless steel surface can be calculated, assuming that (i) the layer formed is at the closest packing, and (ii) packing at the air–water interface (measured by Langmuir trough) is similar to the hydrocarbon/steel interface (quantified by QCM).

The calculated surface coverages of the two model OFMs on stainless steel substrate are presented in Fig. 5b. For the two cases, palmitic acid in hexadecane and pentadecylamine in aromatic diesel, the surface coverage values are over 100%, implying either the OFMs form multi-layers at the solid–liquid interface or that they could pack tighter at the hydrocarbon/steel interface than at the air/water interface. We speculate that the latter is more likely because interactions between water molecules and the acid headgroup of the additive are likely to influence how tightly they can pack together, and there could be more electrostatic effects in water than in hexadecane. The rationale is evidenced by that the calculated surface coverage is significantly lower for palmitic acid in saturated oil (25%) and aromatic oil (60%) when there are more water molecules present (Fig. 1b). This implies that palmitic acid molecules did not form a packed layer that covers the solid surface, hence the adsorbed layer could be highly tilted, which is supported by the interfacial adhesion data (Fig. 5a) where the large values acquired in the complex hydrocarbon imply a tilted molecular orientation in the adsorbed film. Pentadecylamine shows slight differences between all three solvents, but all within a standard deviation of each other ranging from 80 to 100% surface coverage. It suggests that a generally well packed layer pentadecylamine was observed. The limiting area per molecule data (Fig. 5a) also provides evidence of why the impact of water on pentadecylamine is much less than for the palmitic acid: pentadecylamine can pack together much more tightly, countering against the disruption caused by water molecules.

It must be noted that the assumptions for calculating the surface coverage come some limitations: surface coverage of OFM on stainless steel substrate was estimated by dividing the mass adsorbed measured by QCM with the limiting area quantified by Langmuir trough. The Langmuir trough data observed compression at a water–air interface, which is not directly analogous to the steel–hydrocarbon interface being measured in the QCM. In here, the QCM measurements do not account for the potential of solvent interaction with the functional groups of the OFMs, which could cause an increase to the adsorbed mass observed. Therefore, the absolute values of percentages calculated in the present work are estimations. However, this should not underestimate the value of the trends observed with regards the effect of solvent and OFM chemistry.

### Evaluating Additive Hydrocarbon Mixtures at the Macroscale

**Fig. 6:**
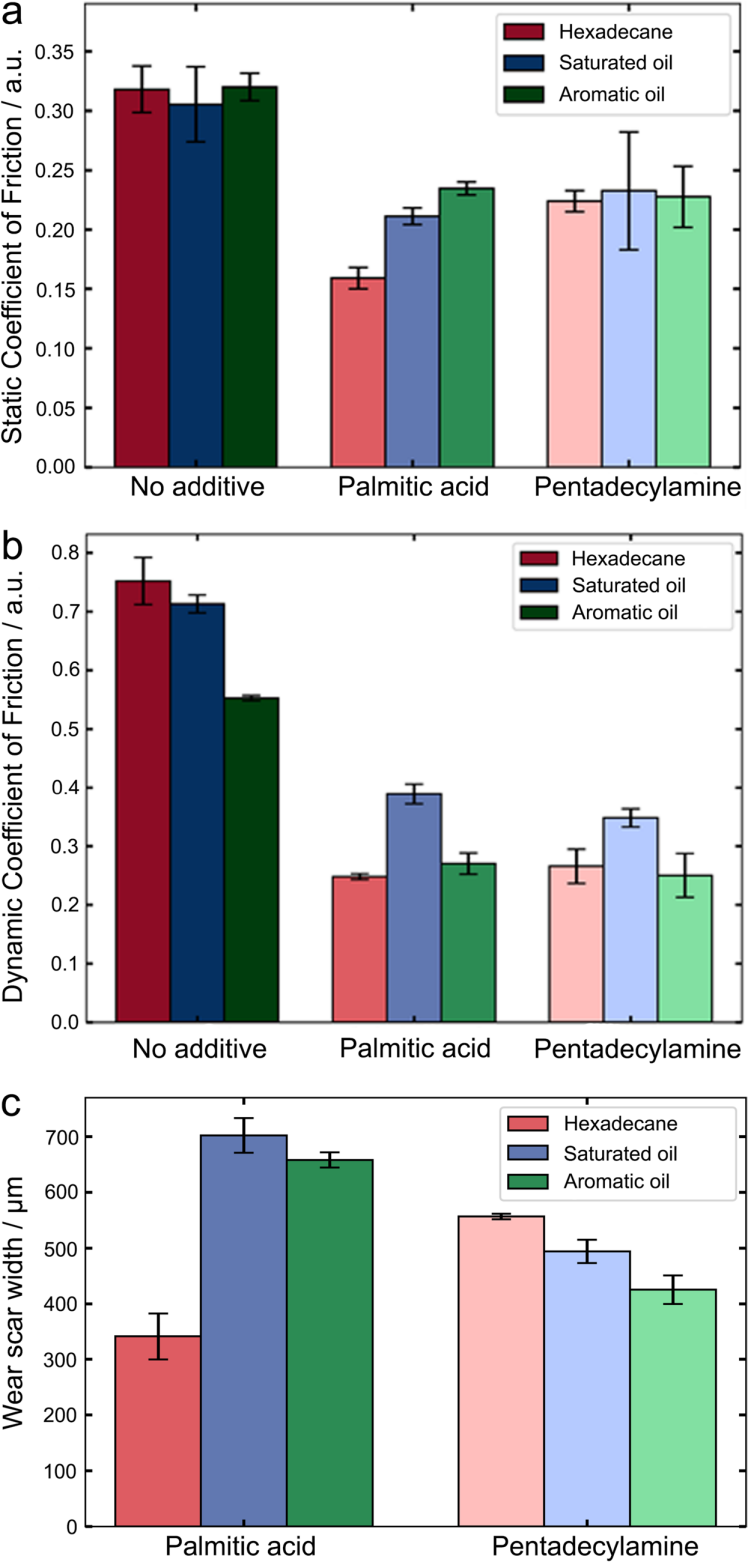
**a** Static COF, **b** dynamic COF, and **c** wear scar width of the two OFMs in three different hydrocarbon samples

With knowledge of the molecular characteristics, such as surface binding, packing, kinetics, and friction, it is critical to establish how such molecular features could influence the macroscale tribological performance such as dynamic and static COF measured by a wear tester. Figure 6a presents the static COF, the friction required to initiate sliding, acquired with or without the model OFM in three different hydrocarbon. Little difference was observed between the hydrocarbons without OFM, suggesting that the static friction between steel–steel contact is not governed by the liquid at the articulating interface. This is likely because the static COF is determined by the force to overcome the friction in initiating a relative motion, which has little contribution from the liquid within the contact under the boundary lubrication condition.

Once the OFMs were introduced, static friction was found to reduce, suggesting that the surface adsorbed OFM film could reduce the force required to initiate the relative motion. The static COF is lower for palmitic acid in hexadecane than it is in saturated and aromatic oil. Naturally, the static COF would be influenced by factors affecting the film in equilibrium such as the magnitude of the molecular binding of the headgroups, with a stronger molecular binding leading to a better formed film and therefore a lower static COF. The friction of the formed film would also affect the static COF with larger friction of the film contributing more to a larger static COF. But other factors such as the adsorption processes will not have as significant an impact on the static COF. Pentadecylamine shows very little change in the static COF, which is consistent with the observation at single asperity contacts (Fig. 3a), suggesting that static friction is primarily determined by the OFM film formed under thermodynamic equilibrium. The lower the interfacial adhesion force, the lower the static COF.

The dynamic COF values, measured by the rotating wear test, are shown in Fig. 6b. For the hydrocarbon without OFMs, there is a trend correlating the surface tensions of the hydrocarbon (Fig. 1a): the aromatic oil with the highest surface tension resulted in the lowest dynamic COF, likely because it contains more water that could interact with the steel much more favourably compared to the hydrocarbon oils of hexadecane and the saturated oil. Once palmitic acid and pentadecylamine were introduced, their dynamic COF values present a correlation with the interfacial adhesion force (Fig. 2b), with the saturated oil resulting in the largest dynamic COF amongst the hydrocarbon, and hexadecane and aromatic oil both having similarly low dynamic COF. It confirms the relationship between surface adhesive interactions and dynamic COF because dynamic friction, during the articulating motion, is determined by the constant forming and breaking of adhesive interactions. Sliding friction could also be affected by the binding affinity of the OFMs to the surface as the OFM molecules are expected to be removed and re-adsorb to the surface under high normal load. Previous study has been shown that reducing the adhesive interactions at the surface in non-polar solvents can significantly reduce COF values [54].

Wear scar on the steel ball used in the rotating wear tester was imaged by an optical microscope post the macroscale tribological measurements, of which the width is shown in Fig. 6c. For palmitic acid, the wear scar width was the smallest (330 µm) in hexadecane but increased considerably in aromatic oil and saturated oil (around 650 and 700 µm, respectively). A different trend was observed with pentadecylamine that has a high wear scar width in hexadecane, which decreased slightly in saturated oil, and further in aromatic oil. Wear is determined by the ability of the additive film to protect the surface of the steel. The test method used in the present work, akin to HFRR [55], comes with high contact pressures (reaching a maximum Hertzian contact pressure of 1.2 GPa), under which the surface adsorbed OFM film would highly likely be removed constantly upon contact. For such scenario, the OFM’s ability to re-adsorb onto the surface (i.e., adsorption kinetics) is probably of the utmost importance to maintain the protective film.

### Correlating Molecular Characteristics of OFM and the Corresponding Macroscopic Tribological Performance

**Fig. 7:**
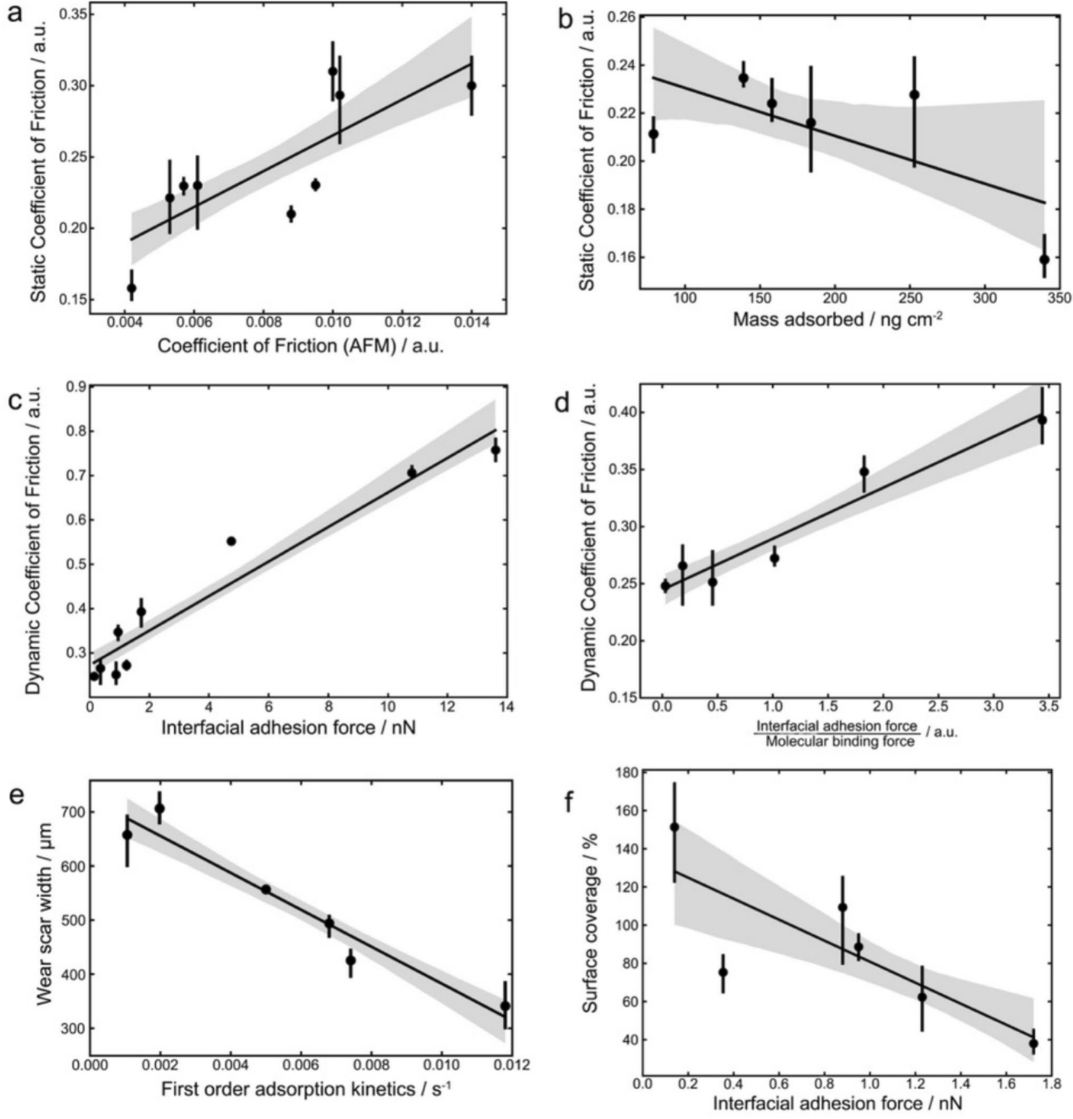
Correlation plots of macroscopic tribological characteristics against molecular properties: **a** Static coefficient of friction vs. COF measured by AFM; **b** Static COF vs. mass adsorbed onto the surface; **c** Dynamic COF vs interfacial adhesion force; **d** Dynamic COF vs interfacial adhesion divided by the molecular binding force; **e** Wear scar width vs the first-order adsorption kinetics; **f** Surface coverage vs interfacial adhesion force

Upon the investigation of molecular characteristics of OFMs and macroscopic tribological measurements, it is imperative to establish the correlation over different length scales. Our results suggest that the interfacial adhesion is related explicitly with the properties of the surface adsorbed film of OFM, whilst the first-order adsorption kinetics underpins the ability of OFM molecules to re-adsorb after being removed after the solid surface during friction testing. The synergistic effect of molecular phenomena can be measured as COF and wear at the macroscopic scale, using benchtop testing rigs. We have been able to establish correlations between parameters at different length scales, which are presented in Fig. 7.

Figure 7a presents the static COF measured by the wear tester and the COF measured at the nanoscale with AFM. Considering that the static COF is the force required to initiate a sliding motion, which is governed by the characteristics of the formed equilibrium film, the positive correlation was well expected. A correlation was also observed between the equilibrium mass adsorbed of the OFMs and the static COF (Fig. 7b), which provides further proof that the static COF is primarily dependent on the properties of the adsorbed film under equilibrium state.

In contrast, dynamic COF depends more on the interfacial phenomena during the articulating motion, such as how strongly the film is bound to the solid substrate, and the interfacial adhesion force as sliding at macroscopic scale would constantly form and break bonds between OFMs and the solid surfaces. Figure 7c presents the dynamic COF, measured by the wear tester, as a function of the interfacial adhesion force quantified by AFM, which shows a positive relationship between the two. It must be noted that the satisfactory linear fitting is dominated by the three largest values of interfacial adhesion force acquired from hydrocarbon with no additives, hence the relationship, in particularly the effect of OFMs, may be less strong.

As discussed earlier, dynamic friction would be determined by both the interfacial adhesive interactions and the quality, such as robustness and surface coverage, of the adsorbed OFM film. To this end, dynamic COF values were related, in Fig. 7d, to an arbitrary parameter calculated by dividing the interfacial adhesion force by the molecular binding for each combination of hydrocarbon and OFM additive. The smaller this parameter, the stronger bound the film to the solid substrate, and less adhesive interactions at the contact interface, supported by the satisfactory fitting. This finding implies that the rapid removal of the adsorbed OFM film from the surface will contribute to a larger sliding friction force when the binding affinity of the OFM to the surface is not adequate, even if the adhesive interactions at the interface are minimised. To implement an excellent interfacial lubrication, the OFM molecules must be able to (i) minimise adhesive interactions at the interface with the tailgroups exposing to the hydrocarbon and (ii) bind strongly to the steel substrate via the headgroup.

Figure 7e shows the wear scar width quantified post macroscale friction testing as a function of the first-order adsorption kinetics based on QCM measurements. The strong correlation suggests that the faster the adsorption kinetics, the less wear observed, which reinforces the knowledge that the extent of an OFM to protect the steel surface is determined by its ability to re-adsorb rapidly once being removed during to interfacial friction. Whilst the wear data does not seem directly linked to any other molecular characteristics of the OFM, we would argue that preventing wear is an integral function of OFM. It is of great importance for the OFM molecules to possess fast surface adsorption kinetics, which would result in a minimal wear [22, 23].

Finally, Fig. 7f shows the relationship between the interfacial adhesion and the surface coverage percentage that was calculated using the molecular area of the OFM at the air/water interface and the equilibrium mass adsorbed quantified by QCM. Even though it is likely that the surface coverage values are overestimated due to the subphase of the trough measurements being water, a connection between surface coverage and interfacial adhesion was observed, which suggests that the interfacial adhesion values, when the OFMs consist of the same tail groups in the present work, relate directly to the packing density of the surface adsorbed film. It reinforces the knowledge that the more loosely packed the film is, the further apart between the adsorbed OFM molecules, the more asperities for contact at the interfaces, and consequently the greater the interfacial adhesion force.

## Conclusion

In the present work, a multi-scale tribological approach was deployed to investigate both the molecular characteristics of OFMs, including binding, film packing, interfacial adhesion, and adsorption kinetics, and the macroscopic frictional performance such as static and dynamic COF and wear scar. Palmitic acid and pentadecylamine were examined in a range of hydrocarbon to investigate the effect of the headgroup of OFM and the solvent quality on the interfacial behaviour, hence the tribological properties.

The effect of solvent on the interfacial properties of palmitic acid is profound, with the least molecular binding and surface coverage in the saturated oil that contains a relatively large fraction of water. It is believed that the water molecules would compete for binding sites available on the solid substrate, hence limit the ability of the palmitic acid to form a strongly bound and tightly packed surface adsorbed film. Although the aromatic oil has a similar quantity of water as the saturated oil, the effect of the water on palmitic acid is less significant. This could be due to the presence of the aromatic compounds that would interact with water molecules in bulk, limiting the water available at the liquid/ steel interface to compete against the OFM.

On the other hand, interfacial behaviour of pentadecylamine is not affected by the solvent as much. We recognise that the exact molecular mechanisms of the OFMs in different hydrocarbons, underpinning their tribological performance, cannot be fully unravelled due to the limited knowledge concerning the chemical compositions and the complexity of the commercial hydrocarbon used. However, the correlations identified in the present work show that the hydrocarbon quality and composition play a critical role in the overall performance of the OFM, such as surface binding, surface packing, and interfacial friction. The compression isotherms show that pentadecylamine is less disrupted, in comparison to palmitic acid, by the presence of water. In hexadecane where minimal amount of water was observed, palmitic acid was able to introduce the greatest lubrication and minimal wear, highlighting the importance of solvent composition and quality on the performance of the OFMs.

Correlations between the molecular characteristics of OFMs and their corresponding macroscopic frictional results show that

- Static friction is determined by the properties of the OFM film formed at equilibrium state, which is consistent with the previous studies. The mass of the OFM molecules adsorbed in equilibrium correlates well with the COF measured on the nanoscale of the adsorbed film, which confirms the importance of having a well-structured and densely packed OFM film at the solid/liquid interface in providing lubrication.
- It must be emphasised that dynamic friction is determined by the interactions between the two surfaces in contact and the affinity of the OFMs to the solid substrate, which is evidenced by the strong correlation between the dynamic COF and the arbitrary parameter that relates interfacial adhesion force and molecular binding of the OFMs to the surface. This suggests that dynamic COF could be minimised by reducing the adhesive interactions between the tailgroup of OFM and the counter surface, and by maximising the binding affinity between the headgroup of OFM and the solid substrate.
- We would like to highlight that wear from the macroscale analysis is correlated to the first-order adsorption kinetics of the additives determined from QCM, which reinforces the importance of the ability to quickly adsorb to the solid surface for OFM in reducing wear. As the surface adsorbed OFM molecules are constantly being removed and re-adsorbing, the kinetics of such re-adsorption is critical for wear reduction.

## Conflict of interest

The authors declare no competing interests.

## Data Availability

All data supporting the findings of this study are available within the paper.

## References

[1] Langmuir, I.: The Mechanism of the Surface Phenomena of Flotation. Transac. Faraday Soc. **15**(June), 62–74 (1920)

[2] Bowden, F.P., Gregory, J.N., Tabor, D.: Lubrication of metal surfaces by fatty acids. Nature **156**, 97–101 (1945)

[3] Bowden, F.P., Leben, L.: The friction of lubricated metals. Proc. R. Soc. A **239**, 1–27 (1940)

[4] Bowden, F.P., Leben, L.: The nature of sliding and the analysis of friction. Proc. R. Soc. A **141**, 691–692 (1939)

[5] Hansen, G.A.T., Lee, P.M., Westbrook, S.R., Wilson, G.R.: Determining the sensitivity of fuel lubricity additive concentration on HFRR test parameters. Mater. Perform. Charact. **7**, 374 (2018)

[6] Delgado, J., Gadea, M., Esarte, C., Peláez, A.: HFRR and SL-BOCLE lubricity of paraffinic diesel fuels considering different origins and final formulations with biodiesels and additives. Energy Fuels **34**, 2654–2664 (2020)

[7] Lacey, P.I., Mason, R.L.: Fuel lubricity: Statistical analysis of literature data. SAE Tech. Pap. Ser. (2000). 10.4271/2000-01-1917

[8] Lehto, K., Vepsäläinen, A., Kiiski, U., Kuronen, M.: Diesel fuel lubricity comparisons with HFRR and Scuffing Load Ball-on-Cylinder Lubricity Evaluator methods. SAE Int. J. Fuels Lubr. **7**, 842–848 (2014)

[9] Kuronen, M.A., Kiiski, U., Lehto, K.: Diesel fuel lubricity comparisons with HFRR and scuffing load ball-on-cylinder lubricity evaluator methods, part II. SAE Tech. Pap. Ser. (2015). 10.4271/2015-24-2498

[10] Spikes, H.: Friction modifier additives. Tribol. Lett. **60**, 5 (2015)

[11] Tartaglino, U., Sivebaek, I.M., Persson, B.N.J., Tosatti, E.: Impact of molecular structure on the lubricant squeeze-out between curved surfaces with long range elasticity. J. Chem. Phys. **125**, 014704 (2006) 16863321 10.1063/1.2210008

[12] He, X., Lu, J., Desanker, M., Invergo, A.M., Lohr, T.L., Ren, N., Lockwood, F.E., Marks, T.J., Chung, Y.-W., Wang, Q.J.: Boundary lubrication mechanisms for high-performance friction modifiers. ACS Appl. Mater. Interfaces **10**, 40203–40211 (2018) 30396273 10.1021/acsami.8b11075

[13] Lundgren, S.M., Persson, K., Kronberg, B., Claesson, P.M.: Adsorption of fatty acids from alkane solution studied with quartz crystal microbalance. Tribol. Lett. **22**, 15–20 (2006)

[14] Wood, M.H., Welbourn, R.J.L., Charlton, T., Zarbakhsh, A., Casford, M.T., Clarke, S.M.: Hexadecylamine adsorption at the iron oxide − oil interface. Langmuir **29**, 13735–13742 (2013) 24106786 10.1021/la4018147 PMC3850247

[15] Karpovich, D.S., Blanchard, G.J.: Direct measurement of the adsorption kinetics of alkanethiolate self-assembled monolayers on a microcrystalline gold surface. Langmuir **10**, 3315–3322 (1994)

[16] Zachariah, Z., Nalam, P.C., Ravindra, A., Raju, A., Mohanlal, A., Wang, K., R., V.C., Espinosa-Marzal, R.M.: Correlation between the adsorption and the nanotribological performance of fatty acid‑based organic friction modifiers on stainless steel. Tribol. Lett. **68**, 11 (2020)

[17] Greenfield, M.L., Ohtani, H.: Friction and normal forces of model friction modifier additives in simulations of boundary lubrication. Mol. Phys. **117**, 3871–3883 (2019)

[18] Fry, B.M., Moody, G., Spikes, H.A., Wong, J.S.S.: Effect of surface cleaning on performance of organic friction modifiers. Tribol. Trans. **63**, 305–313 (2020)

[19] Bonnaud, P.A., Kinjo, T., Sato, N., Tohyama, M.: Adhesion and structure of lubricant films: molecular simulations of amine-based organic additives in base oil at a model of steel surface. Tribol. Int. **193**, 109449 (2024)

[20] Fry, B.M., Chui, M.Y., Moody, G., Wong, J.S.S.: Interactions between organic friction modifier additives. Tribol. Int. **151**, 106438 (2020) 10.1021/acs.langmuir.9b03668 31941274

[21] Fry, B.M., Moody, G., Spikes, H.A., Wong, J.S.S.: Adsorption of organic friction modifier additives. Langmuir **36**, 1147 (2020) 31941274 10.1021/acs.langmuir.9b03668

[22] Nalam, P.C., Pham, A., Castillo, R.V., Espinosa-Marzal, R.M.: Adsorption behavior and nanotribology of amine-based friction modifiers on steel surfaces. J. Phys. Chem. C **123**, 13672–13680 (2019)

[23] Gao, H., Ewen, J.P., Hartkamp, R., Müser, M.H., Dini, D.: Scale-dependent friction–coverage relations and nonlocal dissipation in surfactant monolayers. Langmuir **37**, 2406–2418 (2021) 33545003 10.1021/acs.langmuir.0c03403

[24] Yu, H., Chen, H., Zheng, Z., Qiao, D., Feng, D., Gong, Z., Dong, G.: Effect of functional groups on tribological properties of lubricants and mechanism investigation. Friction **11**, 911 (2023)

[25] Bonnaud, P.A., Kinjo, T., Sato, N., Tohyama, M.: Molecular simulations of amine-based organic additives at a steel surface: effect of the internal molecular structure on adsorption. Tribol. Int. **201**, 110258 (2025)

[26] Ewen, J.P., Echeverri, S., Morgan, N., Dini, D.: Nonequilibrium molecular dynamics simulations of stearic acid adsorbed on iron surfaces with nanoscale roughness. Tribol. Int. **107**, 264–273 (2017)

[27] Ewen, J.P., Gattinoni, C., Morgan, N., Spikes, H.A., Dini, D.: Nonequilibrium molecular dynamics simulations of organic friction modifi ers adsorbed on iron oxide surfaces. Langmuir **32**, 4450–4463 (2016) 27064962 10.1021/acs.langmuir.6b00586

[28] Jaishankar, A., Jusu, A., Vreeland, J.L., Deighton, S., Pellettiere, J., Schilowitz, A.M.: Adsorption of stearic acid at the iron oxide/oil interface: theory, experiments, and modeling. Langmuir **35**, 2033–2046 (2019) 30624939 10.1021/acs.langmuir.8b03132

[29] Gu, H., Hirayama, T., Yamashita, N., Xu, J., Yamada, M.: Relationship between friction reduction effect and solubility in base oil of organic friction modifiers. Tribol. Int. **202**, 110304 (2025)

[30] Konishi, M., Washizu, H.: Understanding the effect of the base oil on the physical adsorption process of organic additives using molecular dynamics. Tribol. Int. **149**, 105568 (2020)

[31] Kicior, I., Matthews, L., Britton, A.J., Willneff, E.A., Hammersley, C., Morina, A., Dowding, P.J., Honkimäki, V., Schroeder, S.L.: Friction and wear reduction by glycerol oleates: the molecular basis for performance variations in the presence of water and acetic acid. Tribol. Int. (2025). 10.1016/j.triboint.2025.111140

[32] Shaigan, N., Neill, W.S., Littlejohns, J., Song, D., Lafrance, S.: Adsorption of lubricity improver additives on sliding surfaces. Tribol. Int. **141**, 105920 (2020)

[33] Wang, H., Chen, S., Li, L., Jiang, S.: Improved method for the preparation of carboxylic acid and amine terminated self-assembled monolayers of alkanethiolates. Langmuir **21**, 2633–2636 (2005) 15779923 10.1021/la046810w

[34] Ogletree, D.F., Carpick, R.W., Salmeron, M.: Calibration of frictional forces in atomic force microscopy. Rev. Sci. Instrum. **67**, 3298–3306 (1996)

[35] Tocha, E., Schönherr, H., Vancso, G.J.: Quantitative nanotribology by AFM: a novel universal calibration platform. Langmuir **22**, 2340–2350 (2006) 16489827 10.1021/la052969c

[36] Varenberg, M., Etsion, I., Halperin, G.: An improved wedge calibration method for lateral force in atomic force microscopy. Rev. Sci. Instrum. **74**, 3362–3367 (2003)

[37] Mehra, R.: Application of refractive index mixing rules in binary systems of hexadecane and heptadecane with n-alkanols at different temperatures. J. Chem. Sci. **115**, 147–154 (2003)

[38] Rolo, L.I., Caço, A.I., Queimada, A.J., Marrucho, I.M., Coutinho, J.A.P.: Surface tension of heptane, decane, hexadecane, eicosane, and some of their binary mixtures. J. Chem. Eng. Data **47**, 1442–1445 (2002)

[39] Lee, D.J., Huang, W.H.: Enthalpy-entropy compensation in micellization of sodium dodecyl sulphate in water/methanol, water/ethylene glycol and water/glycerol binary mixtures. Colloid Polym. Sci. **274**, 160–165 (1996)

[40] Das, S., Mondal, S., Ghosh, S.: Physicochemical studies on the micellization of cationic, anionic, and nonionic surfactants in water-polar organic solvent mixtures. J. Chem. Eng. Data **58**, 2586–2595 (2013)

[41] Fregolente, P.B.L., Fregolente, L.V., Maciel, M.R.W.: Water content in biodiesel,diesel, and biodiesel–diesel blends. J. Chem. Eng. Data **57**, 1817–1821 (2012)

[42] Zhang, L.-J., Zhang, Y., Zhang, R.-J., Feng, X.-S.: In situ AFM investigations on degradation of self-assembled monolayers on mica: effect of humidity. Colloids Surf. A Physicochem. Eng. Aspects **293**, 195–200 (2007)

[43] Briscoe, B.J., Evans, D.C.B.: The shear properties of Langmuir-Blodgett layers. Proc. R. Soc. Lond. A Math. Phys. Sci. **380**, 389–407 (1982)

[44] Gao, J., Luedtke, W.D., Gourdon, D., Ruths, M., Israelachvili, J.N., Landman, U.: Frictional forces and Amontons’ law: from the molecular to the macroscopic scale. J. Phys. Chem. B **108**, 3410–3425 (2004)

[45] Popova, E., Popov, V.L.: The research works of Coulomb and Amontons and generalized laws of friction. Friction **3**, 183–190 (2015)

[46] Richardson, D.E.: Review of power cylinder friction for diesel engines. J. Eng. Gas Turbines Power **122**, 506–519 (2000)

[47] Shoba, T.; Crua, C.; Heikal, M. R.; Gold, M. R. (2011) Optical Characterisation of Diesel, RME and Kerosene Sprays by Microscopic Imaging. In *24th European Conference on Liquid Atomization and Spray Systems*,

[48] Hirayama, T., Kawamura, R., Fujino, K., Matsuoka, T., Komiya, H., Onishi, H.: Cross-sectional imaging of boundary lubrication layer formed by fatty acid by means of frequency-modulation atomic force microscopy. Langmuir **33**, 10492–10500 (2017) 28960989 10.1021/acs.langmuir.7b02528

[49] Sauerbrey, G.: Verwendung von Schwingquarzen zur Wägung dünner Schichten und zur Mikrowägung. Z. Physik **155**, 206–222 (1959)

[50] Reviakine, I., Johannsmann, D., Richter, R.P.: Hearing what you cannot see and visualizing what you hear: interpreting quartz crystal microbalance data from solvated interfaces. Anal. Chem. **83**, 8838–8848 (2011) 21939220 10.1021/ac201778h

[51] Liu, Y., Shen, L.: From Langmuir kinetics to first- and second-order rate equations for adsorption. Langmuir **24**, 11625–11630 (2008) 18788769 10.1021/la801839b

[52] Partyka, S., Zaini, S., Lindheimer, M., Brun, B.: The adsorption of non-ionic surfactants on a silica gel. Colloids Surf. **12**, 255–270 (1984)

[53] Jurak, M., Szafran, K., Cea, P., Martín, S.: Analysis of molecular interactions between components in phospholipid-immunosuppressant-antioxidant mixed Langmuir films. Langmuir **37**, 5601–5616 (2021) 33915045 10.1021/acs.langmuir.1c00434 PMC8280729

[54] Burgess, D., Li, N., Rosik, N., Fryer, P.J., McRobbie, I., Zhang, H., Zhang, Z.J.: Surface-grafted Poly(ionic liquid) that lubricates in both non-polar and polar solvents. ACS Macro Lett. **10**, 907–913 (2021) 34306821 10.1021/acsmacrolett.1c00174 PMC8296680

[55] Hornby, B., Cuckston, G., Caprotti, R., More, I.: Pushing the boundaries of the HFRR: Impact of increased test severity on wear. SAE Tech. Pap. **11**, 2013-2001-2688 (2013)

